# Roles of Cyclic Nucleotide Phosphodiesterases in Signal Transduction Pathways in the Nematode *Caenorhabditis elegans*

**DOI:** 10.3390/cells14151174

**Published:** 2025-07-30

**Authors:** Kranti K. Galande, Rick H. Cote

**Affiliations:** Department of Molecular, Cellular and Biomedical Sciences, University of New Hampshire, Durham, NH 03824, USA; kranti.galande@unh.edu

**Keywords:** cyclic nucleotide signaling pathways, cAMP, cGMP, cyclic nucleotide phosphodiesterase, *C. elegans*, nematode, phosphodiesterase, review

## Abstract

Cyclic nucleotide signaling pathways play essential roles in the physiology of the nematode *Caenorhabditis elegans*, influencing processes such as reproduction, environmental sensing, and cellular homeostasis. The intracellular levels of cAMP and cGMP are tightly regulated by their synthesis by adenylyl and guanylyl cyclases and their degradation catalyzed by 3′,5′-cyclic nucleotide phosphodiesterases (PDEs). Mammals possess eleven PDE families (PDE1 through PDE11), whereas nematode genomes contain six PDE genes orthologous to six of the mammalian PDE families. Despite their evolutionary conservation, the signaling pathways, regulatory mechanisms, and enzymatic properties of nematode PDEs remain incompletely understood. This review synthesizes current knowledge on the regulation of cyclic nucleotide levels in *C. elegans*, highlighting how dysregulation of nematode PDEs affects a wide range of physiological and behavioral processes, including sensory transduction, development, and locomotion.

## 1. Introduction

### 1.1. PDEs and Cyclases Constitute a Metabolic Pathway Responsible for Regulating Cyclic Nucleotide Levels in Cells

**Figure 1:**
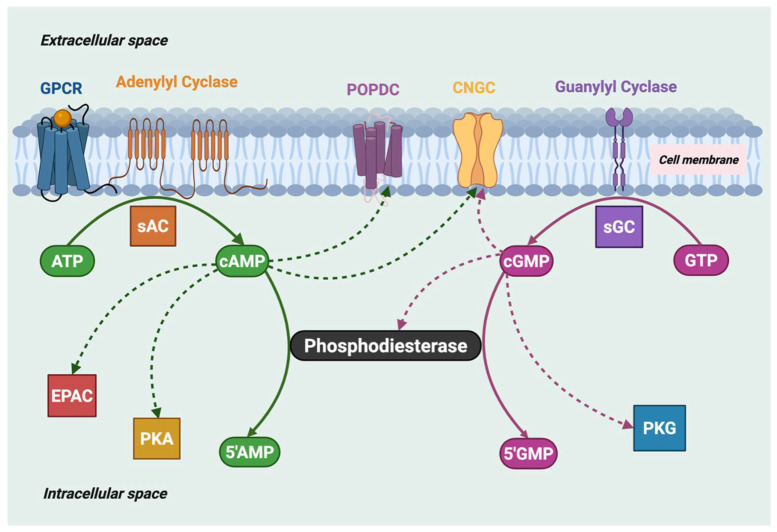
Cyclic nucleotide signaling pathways in eukaryotes: cAMP and cGMP levels are determined by the balance of their synthesis by both soluble adenylyl and guanylyl cyclases (sAC, sGC) and membrane-confined cyclases, and their hydrolysis by cyclic nucleotide phosphodiesterases. The downstream targets that bind cyclic nucleotides to ultimately generate the physiological response are shown: EPAC (exchange protein activated by cAMP), PKA (protein kinase A), POPDC (popeye domain-containing proteins), PKG (protein kinase G), and CNGC (cyclic nucleotide-gated ion channels). The dashed arrow from cGMP to PDE indicates allosteric binding of cGMP to GAF-containing PDEs.

Organisms from all domains of life use cyclic nucleotide signaling pathways to coordinate cellular responses through extracellular and intracellular signals. Cyclic nucleotide phosphodiesterases (PDEs) are ubiquitous enzymes that break down 3′,5′-cyclic adenosine monophosphate (cAMP) and 3′,5′-cyclic guanosine monophosphate (cGMP) and play essential roles in the cyclic nucleotide signaling pathways in cells in coordination with the enzyme families responsible for cAMP and cGMP synthesis, namely adenylyl cyclases (AC) and guanylyl cyclases (GC; Figure 1). Class I PDEs are the only enzyme family in eukaryotes able to catalyze the hydrolysis of 3′,5′-cyclic monophosphates [1].

### 1.2. The PDE Superfamily

The class I PDE superfamily in mammals consists of eleven PDE families (PDE1 to PDE11) that all contain a conserved catalytic domain (PDEase_catalytic_dom; IPR002073) but differ in their sequence homology, sensitivity to inhibitors, three-dimensional structures, kinetic properties, modes of regulation, and intracellular localization [2]. Most mammalian PDE families contain several PDE genes (e.g., PDE1A, PDE1B, and PDE1C) as well as multiple splice variants, resulting in over 100 different isoforms [3]. Some PDE families are primarily responsible for specifically hydrolyzing cAMP (PDE4, PDE7, and PDE8) or cGMP (PDE5, PDE6, and PDE9), whereas the other PDE families have similar activities for both cyclic nucleotides (PDE1, PDE2, PDE3, PDE10, and PDE11) [4].

In humans, most cell types express more than one PDE family, although the relative amounts and subcellular localization or compartmentation of each PDE family may differ [5]. Furthermore, many PDE families have a broad tissue distribution [2], although in a few cases the expression of a single PDE family may be confined to a single tissue/cell type to perform a specialized role [e.g., PDE6, the central effector of phototransduction, is almost exclusively expressed in retinal rod and cone photoreceptors [6]]. The co-expression in individual cells of multiple PDEs regulating the cellular concentrations of both cAMP and cGMP levels (as well as sequestration of PDEs into spatially distinct compartments, “signalosomes” [2]) can make it challenging to attribute a specific physiological role to an individual PDE isoform.

Mutations in the genes encoding PDEs have been identified in many of the PDE families and are associated with a variety of human and rodent diseases [2,7]. Of particular relevance to nematode PDEs, pathologies attributed to mammalian PDEs include: deafness (PDE1C; OMIM: 602987) as well as regulation of olfaction in PDE1C knockout mice [8]; intellectual developmental disorder (PDE2A; OMIM: 602658) as well as embryonic lethality in PDE2A knockout mice [9]; hypertension and brachydactyly syndrome (PDE3A; OMIM: 123805) as well as infertility arising from arrest of oocyte maturation in PDE3A knockout mice [10]; acrodysostosis (PDE4D; OMIM: 600129) as well as impaired growth, fertility, and viability in PDE4D knockout mice [11]; dysregulation of steroidogenesis in PDE8A and PDE8B knockout mice [12]; and dyskinesia and striatal degeneration (PDE10A; OMIM: 610652) as well as altered striatal function and behavioral phenotypes in PDE10A knockout mice [13].

Furthermore, there is evidence that genetic or pharmacological disruptions of the activity of one member of the PDE superfamily can sometimes be partially compensated for by up-regulation of the activity of a member of another PDE family [7]. This may account for the observation that in animal models, the genetic disruption of the activity of one PDE family member is seldom lethal. Disrupting the activity of an individual PDE confined to a nanodomain [2] could also result in “leakage” of cyclic nucleotides and their hydrolysis by a different PDE that normally would not act on this pool of cyclic nucleotides.

### 1.3. The Nematode Phylum Contains Six PDE Families Orthologous to Mammalian PDEs

**Figure 2:**
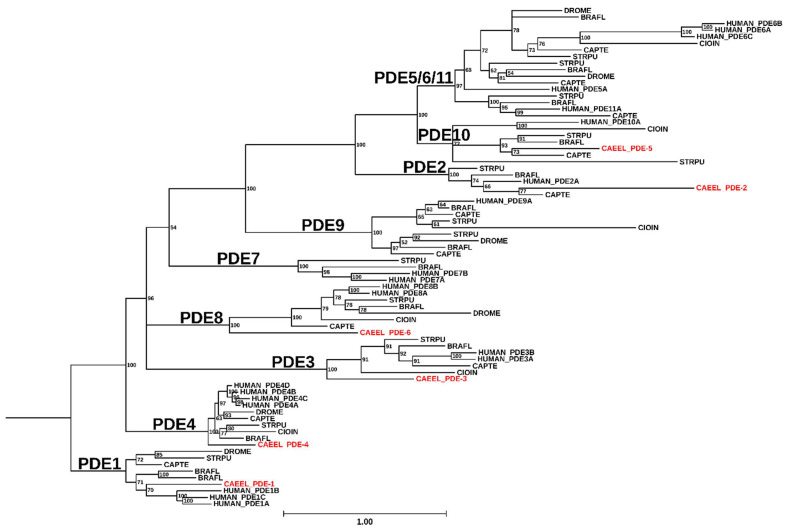
Phylogenetic analysis of *C. elegans* PDEs. All protein sequences annotated with the GO term GO:0004114 (3′,5′-cyclic-nucleotide phosphodiesterase activity) for *Caenorhabditis elegans* (CAEEL), *Homo sapiens* (HUMAN), *Branchiostoma floridae* (BRAFL), *Capitella teleta* (CAPTE), *Ciona intestinalis* (CIOIN), *Drosophila melanogaster* (DROME), and *Strongylocentrotus purpuratus* (STRPU) were downloaded from UniProt [17] on January 25th, 2024. These sequences were subjected to conserved domain search with RPS-BLAST v2.13.0+ [18] against the Class I PDEase catalytic domain (PF00233) [19]. Results of the RPS-BLAST were used to trim the full protein sequences to the bounds of the PDEase domain. Sequences were removed if the PDEase region was <200 AA in length. The remaining sequences were subjected to clustering at 99% identity using CD-HIT v4.7 [20]. The clustered sequences were then aligned using Clustal Omega v1.2.4 [21]. Maximum likelihood phylogenetic inference was performed with IQ-TREE v1.6.12 [22] run with 1000 ultrafast bootstrap replicates and collapsing branches with less than 50% support. The resulting consensus tree was visualized using TreeViewer v2.1.0 [23] and re-rooted to organize PDE1 into a monophyletic group.

Six PDE families orthologous to six of the eleven mammalian PDE families are found not only in *C. elegans* (Figure 2) but also throughout the nematode phylum [14]. In *C. elegans*, these PDEs were classified as PDE-1 to PDE-6 (Table 1), each of which has a human ortholog corresponding to human PDE1, PDE2, PDE3, PDE4, PDE10 (PDE-5 in *C. elegans*), and PDE8 (PDE-6 in *C. elegans*). *C. elegans* PDE-4 has been shown to be a cAMP-specific PDE [14] and PDE-6 is predicted to also have a strong preference for cAMP over cGMP, whereas PDE-1, PDE-2, PDE-3, and PDE-5 are likely capable of hydrolyzing both cAMP and cGMP, based on homology with the characterized enzymatic properties of their mammalian PDE orthologs [4,15]. Unlike the multi-gene mammalian PDE families, the *C. elegans* genome codes for only a single gene for each of the six nematode PDEs.

**Table 1:** Cyclic nucleotide phosphodiesterases expressed in *C. elegans*. Gene names and isoform counts are based on annotations provided in reference [16]. In most instances, the number of isoforms is based on cDNA data, and protein expression of PDE isoforms in *C. elegans* has not, in most cases, been verified. Designation of vertebrate orthologs is based on Figure 2. Except in the case of PDE-4 [14], substrate specificity was assumed to be the same as for human PDEs [4].

Gene Family	Gene Name	Isoforms	Vertebrate Ortholog	Substrate Preference (Vertebrate)
PDE-1	T04D3.3	2	PDE1	cAMP and cGMP
PDE-2	R08D7.6	2	PDE2	cAMP and cGMP
PDE-3	E01F3.1	10	PDE3	cAMP and cGMP
PDE-4	R153.1	9	PDE4	cAMP
PDE-5	C32E12.2	1	PDE10	cAMP and cGMP
PDE-6	Y95B8A.10	3	PDE8	cAMP

## 2. The Overall Structure of PDEs

**Figure 3:**
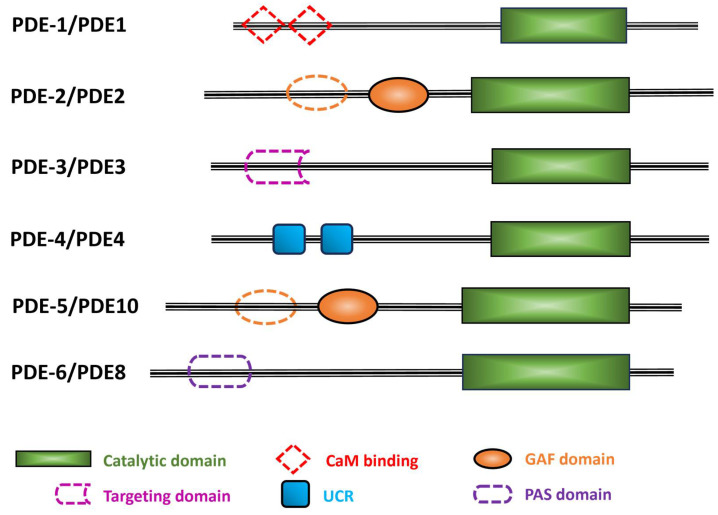
Schematic representation of the six *C. elegans* PDE families. The *C. elegans* PDE family name is indicated with a dash before the numeral, while the human orthologs are in uppercase letters with no space before the numeral. Black lines indicate the relative length of each amino acid sequence. Colored filled shapes indicate the different protein domains that are present in humans and predicted by Uniprot in *C. elegans* PDEs. Dotted shapes are motifs found in human PDEs, but their occurrence in *C. elegans* PDEs is uncertain. Abbreviations used: CaM, calmodulin; GAF, domain found in cGMP phosphodiesterase, bacterial Adenylyl cyclase, and FhlA; UCR, upstream conserved region; PAS, Per-Arnt-Sim.

**Figure 4:**
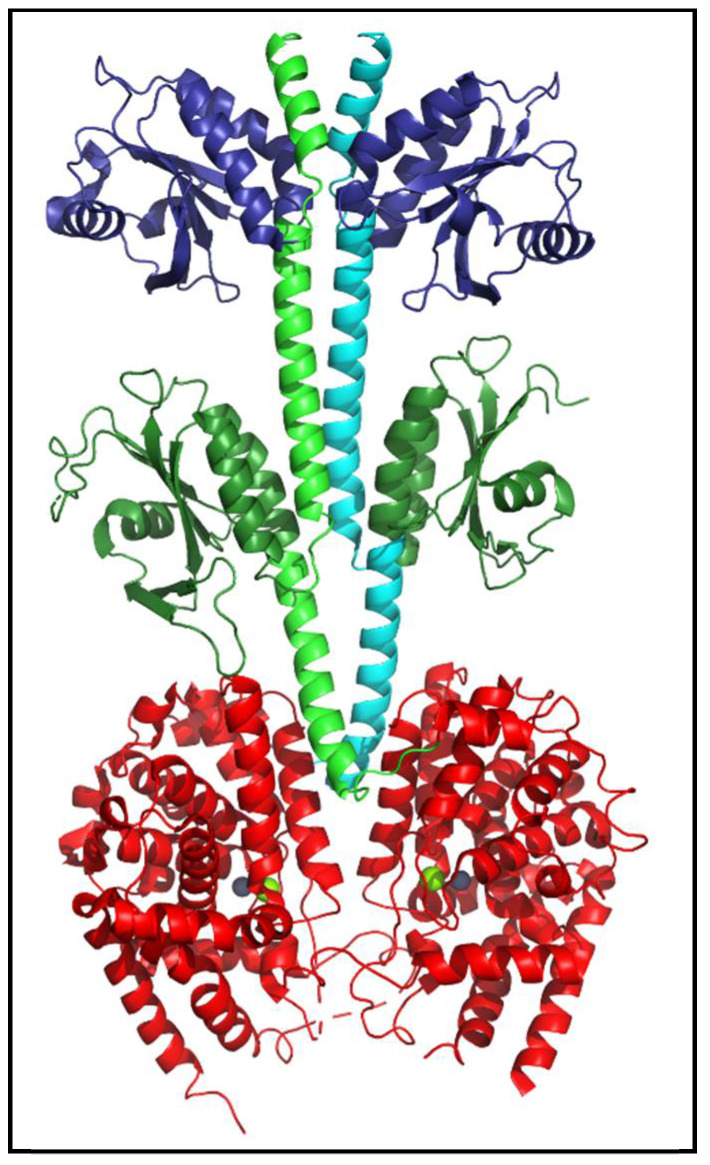
Structure of human PDE2 enzyme. The atomic-level structure of nearly full-length human PDE2 (PDBID: 3IBJ) is a homodimer (reflected in the green and cyan backbone helices of the two subunits). Each catalytic subunit contains two regulatory tandem GAF domains (dark blue, dark green), a catalytic domain (red) containing bound Zn^2+^ and Mg^2+^ (gray and green spheres) at the substrate binding site, and N- and C-terminal regions that were not resolved and thus absent from the depicted structure [25].

PDEs share a general structural organization consisting of an N-terminal region, a regulatory domain, a catalytic domain, and a C-terminal region [Figure 3; [4,24]]. While the structures of isolated regulatory or catalytic domains have been determined at atomic-level resolution for most of the human PDE families, nearly full-length structures have been resolved for mammalian PDE2 [Figure 4; [25]] and PDE6 [6,26]. No *C. elegans* PDE structures have been solved to date.

### 2.1. Catalytic Domain Structure of PDEs

The class I PDE superfamily contains a conserved catalytic domain (PDEase_catalytic_dom; Interpro IPR002073) of ~330 amino acids (Figure 3, green box) with a sequence identity of 35% to 50% among different human PDE families, including 17 residues that are invariant in all 11 PDE families. All six *C. elegans* PDEs have the same invariant residues (with the sole exception of a neutral substitution of alanine for serine in *C. elegans* PDE-2; see Table 2).

The catalytic domain (Figure 4, red) is made up of ~16 α-helices [3,27]. The enzyme active site is formed at the junction of several α-helical residues that are highly conserved among all PDEs. The conserved catalytic domain is essential for substrate-binding, metal-ion binding and catalysis of cyclic nucleotides [4,27]. The substrate binding pocket consists of two consensus metal binding domains (Figure 4) occupied by Zn^2+^ (gray sphere) and Mg^2+^ (green sphere) [4,27].

### 2.2. Regulatory Domains in the Mammalian PDE Superfamily

Different regulatory domains permit each mammalian PDE family to respond to specific regulatory signals, including allosteric modulators, binding sites for effectors, sites undergoing covalent modifications, dimerization domains, autoinhibitory domains, motifs for specific protein/protein interactions, etc. In humans, five PDE families (PDE2, PDE5, PDE6, PDE10, and PDE11) contain two tandem GAF domains (GAFa and GAFb); the functions of the GAF domains include dimerization of the catalytic subunits and cyclic nucleotide-mediated allosteric regulation [28]. Human PDE1 is the only PDE family that contains Ca^2+^/calmodulin (CaM) binding sites, and it is activated by the increase in the cellular concentration of Ca^2+^ [29,30,31]. Human PDE3 has a transmembrane domain as well as several sites of phosphorylation that regulate catalytic activity [32]. Human PDE4 has upstream conserved regions (UCR1) and (UCR2) in its N-terminal region of several isoforms of this multi-gene family that are subject to regulation by reversible phosphorylation [33]. The N-terminal regulatory domain of human PDE8 contains both Receiver (REC) and Per-Arnt-Sim (PAS) domains of uncertain regulatory significance [34,35]. The REC domain receives signals from the sensor component in the 2-component signal transduction system, and the PAS domain is involved in PDE8 regulation, protein-protein interactions, and the binding of small ligands [3,36,37]. PDE7 and PDE9 have no known specific protein domains other than the PDE catalytic domain [38].

## 3. Biochemical Properties and Physiological Roles of *C. elegans* PDEs

In this section, we examine the biochemical properties of each of the six *C. elegans* PDEs, as well as the cyclic nucleotide signaling pathways and physiological processes that can be attributed to an individual PDE family member. In Section 4, we will discuss the complex interplay of multiple PDEs participating in the regulation of *C. elegans* physiology and behavior.

### 3.1. C. elegans PDE-1

The *pde-1* gene (T04D3.3) in *C. elegans* is orthologous to mammalian PDE1 (Figure 2). The human PDE1 family consists of three genes: PDE1A, PDE1B, and PDE1C [31], whereas *C. elegans* has a single *pde-1* in its genome. The ability of *C. elegans* PDE-1 to hydrolyze cGMP and cAMP is inferred from its sequence similarity to the human PDE1 enzyme family. *C. elegans* PDE-1 is activated by Ca^2+^ (as is mammalian PDE1) and degrades cGMP in response to a rise in calcium concentration [39]. In vitro studies have shown that *C. elegans* PDE-1 binds to Ca^2+^/calmodulin [40]; however, sequence divergence of human and *C. elegans* PDE-1 prevents conclusive identification of a calmodulin binding motif in the *C. elegans* PDE-1 amino acid sequence.

#### 3.1.1. Studies Implicating *C. elegans* PDE-1 in Nematode Physiology and Behavior

Chemosensation. In *C. elegans*, acute exposure to CO_2_ elicits an avoidant locomotory response. The BAG sensory neurons are responsible for the detection of CO_2_ gas and utilize a cGMP signaling pathway that includes the receptor-type guanylyl cyclase GCY-9 as the CO_2_ sensor and cyclic nucleotide-gated ion channels to generate the electrical response [41]. PDE-1 is expressed in BAG neurons [39,41], and the ankyrin-repeat scaffold protein, ARCP-1, is responsible for co-localizing PDE-1 and GCY-9 to cilia on the dendritic ends of the neuron [42], thereby forming a localized cGMP signaling microdomain. Disruption of PDE-1 activity results in enhanced turning behavior in response to carbon dioxide [42].

Changes in cGMP levels by the coordinated action of GCY-9 and PDE-1 are also responsible for transcriptional regulation of expression of the gene encoding the neuropeptide FLP-19 in BAG neurons [43]. cGMP-dependent transcriptional regulation of neuropeptide expression is believed to confer behavioral plasticity, given that CO_2_ responses in *C. elegans* are dependent on previous environmental exposure to O_2_ [44]. Taken together, these findings support a role for PDE-1 in feedback regulation following activation of the GCY-9 carbon dioxide sensor that elevates cGMP levels and opens cGMP-gated ion channels. The resulting calcium influx is hypothesized to activate the Ca^2+^/calmodulin regulated PDE-1 enzyme to restore cGMP levels in the dendrites of BAG neurons to their pre-stimulated state.

*C. elegans* is highly sensitive to the concentration of oxygen in its environment, with changes in locomotive behavior in response to either hypoxia or hyperoxia. cGMP is believed to be the primary second messenger in the oxygen-sensing PQR neurons resulting from oxygen binding to the soluble guanylate cyclase that leads to opening of cyclic nucleotide-gated channels (TAX-2 and TAX-4) [45]. Genetic disruption of *pde-1* resulted in a prolonged elevation of cGMP levels in the PQR oxygen-sensing neuron, following an increase in oxygen concentration. Over-expression of the wild-type *pde-1* gene to rescue the defective *pde-1* mutant accelerated the recovery of cGMP levels following excitation [39]. While this observation implicates PDE-1 in negative feedback regulation of the oxygen-sensing signaling pathway within PQR neurons, these authors present additional evidence that supports an interplay of PDE-1 and PDE-2 in overall regulation of the chemosensory mechanisms within the PQR neuron that underlie transduction of changes in oxygen concentration into changes in locomotory behavior [39].

Thermosensation.*C. elegans* is exquisitely sensitive to changes in temperature, and it has been established that several sensory neurons participate in thermotactic behavior. The AFD neurons play an important role in detecting temperature gradients in the nematode’s environment that result in thermotaxis [46,47,48]. In AFD neurons, thermotaxis in response to shallow temperature gradients was disrupted in a *C. elegans* strain harboring a *pde-1* mutation that was cultivated at 15 °C (but lacked a phenotype when cultivated at 20 °C). However, single mutations to *pde-2*, *pde-3*, and *pde-5* also behaved similarly to the *pde-1* mutant [49].

In ASJ neurons, disruption of PDE-1 activity is associated with enhanced cold tolerance; however, a similar degree of enhanced cold tolerance is also observed with *pde-2* and *pde-5* single mutants [50]. These results suggest that multiple PDEs may participate in the regulation of cGMP signaling pathways in these two thermosensitive neurons.

Development. Although most studies involving *C. elegans* PDE-1 implicate this enzyme in cGMP signaling pathways, this dual substrate enzyme has also been reported to be involved in regulating the cAMP signaling pathway responsible for synaptogenesis of motor neurons [51].

#### 3.1.2. Effects of Compounds That Selectively Inhibit Human PDE1 on *C. elegans* In Vivo

Exposure of *C. elegans* in liquid culture to vinpocetine was shown to significantly reduce nematode fat content [52], as well as modestly increase nematode lifespan [53]. Exposure of *C. elegans* to a combination of vinpocetine and zaprinast (a PDE5/6 inhibitor [54] that also inhibits PDE1 at higher concentrations) had an opposite effect on nematode lifespan [55]. It should be noted that both vinpocetine and zaprinast are not highly potent or selective inhibitors of human PDE1, and thus off-target effects cannot be ruled out.

### 3.2. C. elegans PDE-2

The *pde-2* gene (R08D7.6) in *C. elegans* is orthologous to mammalian PDE2A (Figure 2). The substrate specificity of *C. elegans* PDE-2 has not been experimentally determined, but the high degree of sequence similarity within the catalytic domain [39] suggests that nematode PDE-2 is a dual-specificity enzyme capable of hydrolyzing both cAMP and cGMP, as is the case for mammalian PDE2 (Table 1). Unlike mammalian PDE2, the regulatory domain of *C. elegans* PDE-2 is predicted to contain only a single GAF domain; the location of this GAF domain is in proximity to the catalytic domain [39], analogous to the second (GAFb) domain of human PDE2. Since the GAFb domain of human PDE2 is the site for noncatalytic cGMP binding [56] that results in stimulation of catalytic activity [57], *C. elegans* PDE-2 activity is predicted to be regulated in a similar manner. This supports a role for PDE-2 in negative feedback regulation of cGMP levels elevated by stimulation of guanylyl cyclase activity.

#### 3.2.1. Role of *C. elegans* PDE-2 in Nematode Physiology and Behavior

Thermosensation. The signal transduction pathway responsible for thermosensation in AFD neurons is initiated by activation of three different AFD-specific guanylyl cyclases (GCY-8, GCY-18, and GCY-23). The resulting elevation of cGMP levels opens cGMP-gated ion channels, generating thermoreceptor currents [58]. Of these three receptor guanylyl cyclases, GCY-8 plays a dominant role in thermotransduction in AFD neurons [49]. Mutations in the *pde-2* gene resulted in prolonged thermoreceptor currents and elevation of the threshold for activation of the thermosensory pathway in isolated AFD neurons; in addition, the *pde-2* mutant failed to adapt to changes in the “holding” temperature [49]. These results support a model in which PDE-2 is required for the recovery and adaptation phases of the cGMP thermotransduction cascade in AFD neurons.

The ASJ neuron responds to multiple types of stimuli, including light, pheromones, and temperature (specifically cold tolerance), and is reported to express PDE-1, PDE-2, PDE-3, and PDE-5 [50,59]. A mutation in the *pde-2* gene causes abnormal cold tolerance [50], but the absence of a strong phenotype in this *pde-2* mutant makes it challenging to discriminate the relative importance of PDE-2 compared with the other cGMP-hydrolyzing PDEs in regulating cold tolerance in ASJ neurons [59,60].

Development. In addition to sensing the environment, sensory neurons in *C. elegans* also regulate downstream pathways involved in *C. elegans* development and behavior [61]. It has been demonstrated that a *pde-2* mutant in which the GAF and catalytic domains were deleted is defective in normal body size regulation; exposure of *C. elegans* to interfering RNA directed to *pde-2* also disrupted body size regulation [62]. In addition, the *pde-2* mutant exhibited reduced retention of eggs in the uterus compared with the wild-type strain [62].

The signaling pathway regulating body size relies in part on the balance between guanylyl cyclase activity (GCY-12) and PDE-2 activity to determine the intracellular concentration of cGMP [62]. It is likely that the initial elevation of cGMP levels resulting from activation of GCY-12 causes activation of the cGMP-dependent protein kinase (EGL-4) as well as binding of cGMP to the PDE-2 regulatory GAFb domain, which stimulates hydrolysis of cGMP. This may represent a negative feedback mechanism in which PDE-2 activity is stimulated in order to restore cGMP homeostasis to the basal level prior to GCY-12 activation.

#### 3.2.2. Effects of Compounds That Selectively Inhibit Human PDE2 on *C. elegans* In Vivo

Exposure of *C. elegans* to a human PDE2-selective inhibitor (BAY-60-7550) decreased the sensitivity to and delayed the recovery of thermoreceptor currents in response to changes in temperature in isolated AFD neurons, similar to the abovementioned effects of mutating the *pde-2* gene in the AFD neuron [49]. From this, we conclude that PDE2 in humans and *C. elegans* share common interaction sites for the binding of BAY-60-7550, making it a useful pharmacological agent for interrogating PDE-2 function in nematodes.

### 3.3. C. elegans PDE-3

The *C. elegans pde-3* gene (E01F3.1) is an ortholog of mammalian PDE3A and PDE3B (Figure 2), but only a single gene is present in the *C. elegans* genome. Human PDE3 is a dual substrate enzyme, but the substrate specificity of nematode PDE-3 has not been experimentally determined.

#### 3.3.1. Studies Implicating *C. elegans* PDE-3 in Nematode Physiology and Behavior

Thermosensation. A *pde-3* mutant cultivated at 15 °C exhibited abnormal thermotactic responses to shallow thermal gradients, although this *C. elegans* mutant behaved similarly to wild-type when exposed to steeper thermal gradients or when cultivated at 20 °C [49]. Since the expression of *pde-3* was not detected in AFD neurons, this behavioral phenotype is likely arising from a cGMP signaling pathway in other thermosensitive neurons.

Intracellular calcium concentrations in ASJ thermosensory neurons increase on heating and decrease on cooling in *C. elegans*. In a strain harboring a mutation in the *pde-3* gene, the change in calcium concentration upon cooling was weaker compared to the wild type, suggesting that PDE-3 can act as a negative regulator in response to cooling stimuli [59]. However, *pde-1*, *pde-2*, and *pde-5* mutants also exhibit disrupted calcium levels in the ASJ neuron upon warming [59], suggesting that there may be redundancy in the action of cGMP-hydrolyzing PDEs involved in the thermosensory pathway in ASJ neurons.

Chemosensation. Prolonged exposure to volatile odorants has been shown to lead to translocation of the cGMP-dependent protein kinase G (EGL-4) from the cytoplasm to the nucleus of AWC neurons [63]. Over-expression of *pde-3* under the control of a heat-inducible promoter enhances nuclear localization of protein kinase G, supporting the idea that reducing cGMP levels by enhancing PDE-3 activity in the AWC neuron is an element of the olfactory adaptation pathway upon sustained exposure to odorants [64]. However, the same study also reported no defects in olfactory adaptation for the *pde-3* mutant (nor any other single PDE mutant).

Phototransduction. Although nematodes lack eyes, it is well-established that *C. elegans* undergoes avoidance behavior (phototaxis) in response to short-wavelength illumination that activates the LITE-1 receptor [65]. Electrical recordings of the response to light stimulation in isolated ASJ neurons of wild-type and mutant strains have implicated a G-protein-based signaling pathway [66]. Comparison of the photoresponse of PDE quadruple mutants (*pde-1:pde-2:pde-3:pde-5*) with the triple mutant (*pde-1:pde-2:pde-5*) demonstrated that both mutant strains had larger light-induced currents than wild-type nematodes; however, the triple mutant retaining PDE-3 activity had a faster and more complete recovery to the dark-adapted state [66]. This result suggests that PDE-3 may play a non-redundant role in determining the kinetics of photoresponse recovery in ASJ neurons.

#### 3.3.2. Effects of Compounds That Selectively Inhibit Human PDE3 on *C. elegans* In Vivo

The human PDE3 inhibitor trequinsin was found to extend the lifespan of *C. elegans* through enhancing resistance of nematodes to oxidative stress [53].

### 3.4. C. elegans PDE-4

The *C. elegans pde-4* gene (R153.1) is orthologous to mammalian PDE4 (Figure 2) as well as the *Drosophila dunce* gene [67]. Four PDE4 genes exist in the human genome, but only one *pde-4* gene is present in *C. elegans*. However, the *C. elegans pde-4* gene generates multiple isoforms that differ in their N-terminal sequences [16], several of which are likely to be physiologically relevant, analogous to the well-studied splice variants of mammalian PDE4 [68]. Two highly conserved regulatory elements (UCR1 and UCR2) found in human PDE4 isoforms are also present in *C. elegans* PDE-4 [69] (Figure 2), suggesting evolutionary conservation of both the catalytic properties and regulatory mechanisms of this enzyme family.

#### 3.4.1. Studies Implicating *C. elegans* PDE-4 in Nematode Physiology and Behavior

Mechanosensation. A mutation of the *pde-4* gene caused a decrease in the arousal thresholds to body touch during quiescence, as well as reducing the duration of sleep-like quiescence [70]. The mechanism of mechanosensation was further investigated using a *C. elegans* strain in which a glial cell chloride channel (CLH-1) was genetically disrupted, resulting in a nose-touch-insensitive phenotype; disrupting PDE-4 activity that is present in the neighboring ASH sensory neuron or over-expressing adenylyl cyclase (ACY-1) restored touch sensitivity [71]. Further support for the involvement of a cAMP signaling pathway for mechanotransduction was obtained by the observation that a quadruple mutant inhibiting the four cGMP-hydrolyzing PDEs (*pde-1:pde-2:pde-3:pde-5*) did not rescue nose touch insensitivity in this *C. elegans* strain. Conversely, overexpression of *pde-4* in ASH neurons also resulted in insensitivity to nose touches [71]. Taken together, these studies support the notion that the intracellular levels of cAMP (regulated by the balance of ACY-1 and PDE-4 activity) determine the threshold sensitivity for responses to nose touches.

Sleep-like behavior (lethargus) in *C. elegans* occurs before each of the four larval molts and is characterized by a reduction in sensory responsiveness to mechanical or chemical stimuli [72]. A loss-of-function *pde-4* mutant, a gain-of-function *acy-1* mutant, or a loss-of-function mutation in *kin-2* (the regulatory subunit of protein kinase A) have each been shown to enhance sensory responsiveness during lethargus [73,74], supporting the idea that elevation of cAMP levels antagonizes the quiescent state in nematodes.

Locomotion. Studies have shown that mutations in the *pde-4* gene result in a hyperactive phenotype [69,75]. Furthermore, overexpression of *pde-4* in neurons not only rescued the hyperactive phenotype of the mutant strain but also reduced the locomotion rate below that of the wild-type strain [69]. PDE-4 is believed to participate in a G-protein coupled signaling pathway in which cAMP levels in GABAergic motor neurons are regulated by the balance of adenylyl cyclase (ACY-1, which is activated by Gαs (GSA-1)) and PDE-4. Upon activation of adenylyl cyclase activity or disruption of PDE-4 activity, elevated cAMP activates protein kinase A, leading to inactivation of a potassium channel that enhances locomotive activity [75]. In this scenario, the action of PDE-4 likely serves to reduce the locomotion rate following transient elevation of cAMP levels induced by activation of adenylyl cyclase [72,73,74].

Development. The Gαs signaling pathway that regulates cAMP levels through the opposing actions of adenylyl cyclase and PDE has been implicated in *C. elegans* egg-laying behavior, as judged by the observation that a *pde-4* mutant results in hyperactive egg-laying behavior, as does a gain-of-function adenylyl cyclase (*acy-1*) mutant [76]. Interestingly, a gain-of-function mutant of protein kinase G (*egl-4*) also caused hyperactive egg-laying and premature release of embryos [76], implicating the cGMP signaling pathway in egg-laying behavior, although the PDEs participating in cGMP signaling have not been identified.

Cyclic nucleotide signaling pathways play an important role in neuronal development and plasticity in *C. elegans*. The rate and extent of regeneration of axons of the PLM mechanosensory neurons were enhanced in a loss-of-function *pde-4* mutant, suggesting that increased cAMP levels stimulate regeneration of damaged axons and their reconnection. Conversely, overexpression of *pde-4* in these sensory neurons reduced axonal regeneration [77]. Disruption of the regulatory subunit (*kin-2*) of PKA had similar effects to the *pde-4* mutant, implicating PKA as the downstream effector of cellular changes in cAMP levels [77].

It has been shown that elevation of cAMP levels in the DD sub-group of GABAergic motor neurons resulting from reduced transcription of the *pde-4* gene enhanced presynaptic respecification, whereas increased expression of *pde-4* in the VD sub-group of motor neurons lowered cAMP levels and suppressed synaptic respecification [78].

Although absent from the *C. elegans* genome, in mammals the DISC1 (Disrupted in Schizophrenia 1) gene plays an important role in neuronal growth and synapse formation. Transgenic nematodes in which the mouse DISC1 gene was expressed in DD and VD motor neurons led to defects in axon guidance. These defects were rescued by RNAi knockdown of *pde-4* [79]. This further supports the critical role of the cAMP signaling pathway, and specifically PDE-4, in regulating *C. elegans* neuronal development and in neuronal regeneration following injury.

#### 3.4.2. In Vitro Biochemical and Pharmacological Properties of *C. elegans* PDE-4

Recombinant expression of *C. elegans* PDE-4 catalytic domain has confirmed that the nematode PDE4 is a cAMP-specific enzyme with a Km = 1.7 μM, similar to that of human PDE4 [14]. In a *C. elegans* mutant lacking PDE-4 activity, cAMP levels in vivo were elevated in comparison to the wild-type strain [80], consistent with the substrate specificity of *C. elegans* PDE-4 for cAMP determined in vitro.

The *C. elegans* PDE-4 catalytic domain was evaluated for its sensitivity to compounds that are potent inhibitors of human PDE4. Roflumilast (human PDE4-selective inhibitor) and zardaverine (a human PDE3- and PDE4-selective inhibitor) were 159- and 77-fold less effective in inhibiting *C. elegans* PDE-4, likely resulting from differences in the amino acid residues lining inhibitor binding pocket, whereas the non-specific inhibitor 3-Isobutyl-1-methylxanthine (IBMX) bound equally well to both human and nematode enzymes [14].

#### 3.4.3. Effects of Human PDE4 Inhibitors on *C. elegans* In Vivo

The *C. elegans* dnj-14 gene is orthologous to the human DnaJ/Hsp40 family of heat shock proteins, and is involved in lifespan determination and locomotion, The *C. elegans dnj-14* loss-of-function mutant exhibits a shortened lifespan and impaired chemotactic response to isoamyl alcohol; exposure to either resveratrol or rolipram (both PDE4-selective inhibitors) rescued both mutant phenotypes [81]. Administration of rolipram to a transgenic strain of *C. elegans* expressing the mouse DISC1 gene in motor neurons behaved similarly to RNAi knockdown of PDE-4 activity in suppressing defects in axonal guidance [79]. Although several human PDE4 inhibitors have lower affinity for the recombinant *C. elegans* PDE-4 catalytic domain [14], these in vivo studies support the efficacy of human PDE4 inhibitors to be taken up and act on the nematode PDE-4 enzyme.

### 3.5. C. elegans PDE-5 (Human PDE10)

The *C. elegans pde-5* gene (C32E12.2) is orthologous to mammalian PDE10 (Figure 2), but the substrate specificity and pharmacological properties of *C. elegans* PDE-5 have not been evaluated. The amino acid sequence of *C. elegans* PDE-5 is 48% identical to the human PDE10A gene and shows a similar exon organization to human PDE10A [82]. Although all human GAF-PDEs (including PDE10) contain two tandem GAF domains (Figure 4), only the second, GAFb domain of *C. elegans* PDE-5 is reported in Uniprot. Of note is the fact that the GAFb domain is the ligand-binding GAF domain in mammalian PDE10 [83,84], suggesting that allosteric regulation of nematode PDE-5 may be similar to that of human PDE10.

#### Studies Implicating *C. elegans* PDE-5 in Nematode Physiology and Behavior

Thermosensation. As described in previous sections, the cGMP signaling pathway plays an important role in the temperature-sensing AFD neurons that are capable of not only sensing temperature but also memorizing previous temperatures and comparing current and past temperatures [48]. Because several guanylyl cyclases and several cGMP-hydrolyzing PDEs (*pde-1*, *pde-2*, *and pde-5)* are expressed in AFD neurons [49,85], it has been challenging to determine which enzymes participate in the cGMP signaling pathways that respond to temperature stimuli.

A loss-of-function *pde-5* mutant was found to be defective in thermotaxis, and selective expression of the wild-type *pde-5* gene in AFD neurons of the *pde-5* mutant restored normal thermotactic behavior [85]. A *C. elegans* strain containing mutations in both *pde-5* and *pde-1* displayed a stronger phenotype than the *pde-5* mutant alone [85], suggesting possible functional overlap in the action of these two PDEs in the sensory endings of AFD neurons.

Chemosensation. The AWB chemosensory neurons detect volatile chemicals (e.g., 2-nonanone) that typically induce an avoidant response but may also show attractive or avoidant responses to other compounds [86]. For example, low concentration of isoamyl alcohol elicits an attractive response, whereas high concentrations result in avoidant chemotactic behavior [87]. Mutation of the *pde-5* gene was found to reduce the response of AWB neurons to low concentrations of isoamyl alcohol, whereas the *pde-1*, *pde-2*, and *pde-3* single mutants did not affect odorant-induced currents [88]. These results suggest that PDE-5 has a distinct modulatory role in one of the olfactory transduction pathways in AWB neurons that include the following elements: olfactory receptors, Gαi/o subunits (GPA-3 and ODR-3), guanylyl cyclases (ODR-1 and DAF-11), and cyclic nucleotide-gated ion channels (TAX-2, TAX-4, and CNG-3).

### 3.6. C. elegans PDE-6 (Human PDE8)

The *pde-6* gene (Y95B8A.10) in *C. elegans* is orthologous to mammalian PDE8 (Figure 2). Whereas the human genome contains two PDE8 genes, PDE8A and PDE8B, the *C. elegans* genome has only one gene coding for this enzyme. While PDE8 is a cAMP-specific enzyme in humans, the substrate specificity for *C. elegans* PDE-6 has not been experimentally determined. It is uncertain whether *C. elegans* PDE-6 has REC and PAS regulatory domains, as is the case for human PDE8.

#### Studies Implicating *C. elegans* PDE-6 in Nematode Physiology and Behavior

Development. Regulation of cAMP levels by PDE-6 has been implicated in oocyte meiotic maturation and migration of embryos into the uterus [89]. Mutations of the *pde-6* gene have also been implicated in delaying the transit of oocytes from the spermatheca into the uterus; modulation of cAMP levels by PDE-6 occurs in concert with changes in Ca^2+^ signaling to regulate spermathecal contractility responsible for translocating fertilized eggs into the uterus [90]. Of all of the *C. elegans* PDEs, PDE-6 is the least well-characterized nematode PDE, and the signaling pathways in which it participates remain a subject for future investigations.

## 4. Multiple PDEs Coordinately Regulate Some Cyclic Nucleotide Signaling Pathways in *C. elegans*

A number of studies on the role of cyclic nucleotide PDEs in *C. elegans* have relied on introducing mutations into multiple PDE genes in order to detect behavioral or physiological phenotypes that are not observed with disruption of the activity of a single PDE. Global, simultaneous disruption of two or more PDE activities can be challenging when trying to ascribe specific physiological roles to an individual PDE for several reasons: (1) in many cases, isolated mutations in *C. elegans* PDE genes have not been characterized to define the nature of the mutation (i.e., the “molecular phenotype”); (2) whereas the *C. elegans* genome contains two cAMP-specific PDEs (PDE-4 and PDE-6), the remaining four PDEs are likely able to hydrolyze both cAMP and cGMP; (3) based on tissue profiling of PDE gene expression reported in Wormbase [16], most *C. elegans* PDEs are found in multiple cell types, and experimental evidence documenting co-localization of PDEs within a specific cell-type is limited; (4) unlike recent advances in our understanding subcellular compartmentation of cyclic nucleotide signaling in mammalian cells [5], much less attention has been placed on subcellular nanodomains (“signalosomes”) within individual cells in *C. elegans*. With these caveats, we provide several examples from the literature describing the use of *C. elegans* strains harboring mutations in two or more PDE genes in those instances where a strain carrying a single defective PDE gene did not display a strong physiological or behavioral phenotype.

### 4.1. PDE Double Mutants

#### 4.1.1. pde-1:pde-5 and pde-1:pde-2 Double Mutants

PDE-1 and PDE-5 are both present in AFD neurons that regulate thermotactic behavior [49], and both PDEs contribute to the regulation of microvilli-rich neuronal receptive endings [91] where the thermo-sensitive GCY-8 is localized. Mutations in both *pde-1* and *pde-5* result in changes in the morphology of AFD microvilli [91] and disrupt Ca^2+^ signaling dynamics and thermotactic behavior in a temperature-dependent manner [85]. The *pde-1:pde-5* strain also exhibits reduced pruning of AFD neuronal receptive endings by AMsh glial cells [92]. Although the *pde-1:pde-2* mutant strain was found to be defective in thermotactic behavior, comparison with other double mutants suggests that PDE-2 activity in AFD neurons is of secondary importance in regulating cGMP levels [85]. The fact that receptor guanylyl cyclases serve as thermoreceptors in AFD neurons [48,93] supports the idea that PDE-1 and PDE-5 are the primary participants in the thermotransduction pathway in AFD neurons.

#### 4.1.2. pde-2:pde-3 Double Mutant

The *pde-2:pde-3* double mutant was shown to be as effective as a PDE quadruple mutant (*pde-1:pde-2:pde-3:pde-5*) in inhibiting locomotory escape from acute hypoxia due to elevation of cGMP levels, whereas disruption of the two cAMP-specific PDEs (PDE-4 and PDE-6) or the other two dual-substrate PDEs (PDE-1 and PDE-5) was without effect [94]. This result supports the idea that both PDE-2 and PDE-3 regulate the same pool of cGMP responsible for the acute response to hypoxia.

#### 4.1.3. pde-4:pde-6 Double Mutant

Since PDE-4 and PDE-6 are both cAMP-specific enzymes, the *pde4:pde-6* double mutant has been used to evaluate whether changes in cAMP or cGMP concentration are involved in signal transduction pathways in *C. elegans*. For example, the *pde-4:pde-6* double mutant did not alter the light-induced current in ASJ neurons compared to the wild-type photoresponse, in support of the conclusion that the phototransduction pathway in *C. elegans* is a cGMP-dependent signaling pathway [66]. Likewise, isoamyl alcohol-induced responses in AWC neurons were not disrupted when tested in the *pde-4:pde-6* double mutant strain [88], nor was the response to acute hypoxia altered [94].

In contrast, a transgenic strain in which bovine rhodopsin was expressed in *C. elegans* as a reporter system to evaluate phototactic signaling pathways demonstrated that the *pde-4:pde-6* double mutant resulted in a loss of light-induced locomotion mediated by G_i/o_ G-proteins, implicating cAMP as the second messenger for this signaling pathway; conversely, the quadruple mutant of dual-specificity PDEs (*pde-1:pde-2:pde-3:pde-5*) had no effect on light-induced locomotion [95].

### 4.2. PDE Quadruple Mutant

#### pde-1:pde-2:pde-3:pde-5 Quadruple Mutant

Four of the six PDEs are dual specificity enzymes and can hydrolyze both cGMP and cAMP. Reliance on the *pde-1:pde-2:pde-3:pde-5* quadruple mutant in several studies is predicated on the observations that disruption of individual PDEs (Section 3) or subsets of these four dual-substrate PDEs (Section 4.1) often lacked a physiological or behavioral phenotype. As expected, the measured cellular cGMP levels in the *pde-1:pde-2:pde-3:pde-5 mutant* strain were elevated compared to the wild-type strain, whereas cAMP levels were essentially unaltered [80,94]. The phenotypes resulting from the *pde-1:pde-2:pde-3:pde-5* quadruple mutant are discussed below.

Phototransduction. Recording of the photocurrent in ASJ neurons in the *pde-1:pde-2:pde-3:pde-5* quadruple mutant strain revealed a 5-fold stimulation in response amplitude but a greatly slowed, partial recovery to the pre-stimulated state compared with wild-type *C. elegans* [66]. Interestingly, the *pde-1:pde-2:pde-5* triple mutant strain of *C. elegans* had a similar amplitude as the quadruple mutant, but the recovery phase was faster and returned to the pre-stimulated state [66], supporting a role for PDE-3 in photoresponse recovery.

Chemotransduction. Electrical recordings of the AWC neurons of the PDE quadruple mutant (*pde-1:pde-2:pde-3:pde-5*) have revealed that the kinetics (but not the amplitude) of the membrane current upon exposure to low concentrations of isoamyl alcohol are slowed relative to the wild-type strain [88]. In AWB chemosensory neurons, high and low concentrations of isoamyl alcohol induced opposite electrical currents, consistent with behavioral results demonstrating both avoidant responses (to high concentrations of isoamyl alcohol) and attractive behavior (in the presence of low concentrations of isoamyl alcohol) [88]. This suggests that PDEs play a dual role in intensity-dependent responses to isoamyl alcohol.

Environmental alkalinity sensing in the ASEL neuron in *C. elegans* has been shown to require a receptor guanylyl cyclase (GCY-14). Mutants deficient in cGMP-dependent protein kinase (EGL-4), cGMP-gated ion channel (TAX-2 or TAX-4), or the *pde-1:pde-2:pde-3:pde-5* mutant all failed to exhibit a chemotactic response to alkaline pH [96].

The quadruple PDE mutant strain was also tested for its tolerance to oxidative stress and found to have a reduced lifespan compared to wild-type *C. elegans*; this observation was correlated with a substantial elevation of cGMP levels in the mutant strain [80] However, the quadruple mutant strain had a normal lifespan in the absence of environmental stressors [80].

Thermotransduction. The *pde-1:pde-2:pde-3:pde-5* quadruple mutant strain exhibits strong defects in its response to both shallow and steep temperature gradients [49]. The observation of milder defects in thermotaxis in single PDE mutants (described in previous sections) supports the idea that multiple PDEs work in a coordinated and/or redundant manner.

## 5. Conclusions

### 5.1. Synopsis

The *C. elegans* genome encodes six cyclic nucleotide phosphodiesterases (PDEs), each orthologous to a distinct mammalian PDE family: PDE1, PDE2, PDE3, PDE4, PDE8 (designated as nematode PDE-6), and PDE10 (designated as nematode PDE-5). In contrast to mammals where several PDE families comprise multiple genes, each PDE family in *C. elegans* is represented by a single gene. Among these, PDE-4 and PDE-6 exhibit specificity for cAMP, while PDE-1, PDE-2, PDE-3, and PDE-5 are predicted to hydrolyze both cAMP and cGMP. Notably, *C. elegans*—and based on current genomic information, the entire nematode phylum—lack cGMP-specific PDEs such as mammalian PDE5, PDE6, and PDE9. However, existing evidence suggests that the four dual-substrate PDEs in *C. elegans* primarily function in the regulation of intracellular cGMP levels.

Sequence analysis reveals that sixteen of the seventeen invariant residues conserved across the catalytic domains of the eleven mammalian PDE families are also present in *C. elegans* PDEs. This high degree of conservation supports the conclusion that all six nematode PDEs are catalytically active (i.e., not pseudogenes). Biochemical and physiological/behavioral assays further demonstrate that inhibitors selective for human PDEs can disrupt the activity of the *C. elegans* orthologs to PDE-1, PDE-2, PDE-3, and PDE-4, indicating that the structural architecture of the catalytic site of these four PDEs is conserved across species, both in terms of substrate specificity and inhibitor binding. Although the N-terminal regulatory domains of *C. elegans* PDEs show less sequence similarity to their mammalian counterparts, available data suggest that these enzymes are likely regulated through analogous mechanisms.

*C. elegans* offers an unparalleled genetic system for interrogating the physiological and behavioral consequences of disrupting cyclic nucleotide signaling pathways. This literature review highlights the central role cGMP plays as the second messenger for multiple sensory transduction pathways in *C. elegans*. Activation of guanylyl cyclases leads to transient increases in cGMP, which are subsequently attenuated by dual-specificity PDEs that accelerate recovery of cGMP to its pre-stimulated level. Interestingly, single-gene mutations in cGMP-hydrolyzing PDEs seldom result in observable effects on *C. elegans*, and disruption of two or more dual-substrate PDEs is commonly required to detect a physiological or behavioral phenotype. These results are consistent with most cells and tissues expressing multiple PDEs that enable disruption of one PDE to be compensated for by the action of other dual-substrate PDEs. In contrast, cAMP signaling in *C. elegans* appears to be restricted to fewer physiological processes, with roles identified in mechanosensation, locomotion, and developmental processes. Table 3 provides a synopsis of the physiological roles ascribed to each *C. elegans* PDE family member presented in Section 3, along with their cellular localization and the signaling pathway components associated with each nematode PDE.

A comprehensive mechanistic understanding of PDE function in *C. elegans* will require further investigation into the spatial compartmentalization of cyclic nucleotide signaling. Specifically, elucidating the subcellular localization of individual PDEs and their associated signaling components—such as receptors, G-proteins, and cyclases—within nanodomains will be essential. This is particularly important in sensory neurons, where distinct signaling pathways must integrate diverse environmental cues to regulate development and behavior.

### 5.2. Translational Relevance to Human Health and Disease

It is widely accepted that *C. elegans* is an excellent model organism for increasing our understanding of human health and disease [97] [Apfeld and Alper (2018)], based on the majority of its genes having human orthologs, the conservation of biochemical pathways underlying physiological processes (e.g., development, sensory transduction, metabolic regulation, and aging), and the well-established pre-clinical models for human diseases (e.g., neurodegenerative diseases and developmental disorders). This evolutionary conservation of genes and pathways between *C. elegans* and humans is coupled with the cost effectiveness of conducting research on this easily cultured nematode that possesses an extensively curated genome, fully mapped cell lineage and neural connectome, and extensive genetic toolkit (e.g., CRISPR/Cas9, RNA interference, a rich catalog of mutated genes in Wormbase [16]).

Based on the above considerations, research focusing on the role of PDEs in the regulation of cyclic nucleotide signaling pathways in *C. elegans* has direct translational implications for a variety of human diseases involving dysregulation of PDEs (“PDE-opathies”), including disruptions of sensory systems, developmental and reproductive disorders, tumorigenesis, and neurodegenerative diseases (see Section 1.2). Importantly, the ability of family-specific human PDE inhibitors to disrupt the physiology and behavior of *C. elegans* (Section 3.1.2, Section 3.2.2, Section 3.3.2 and Section 3.4.2) should enable high-throughput screening of potential PDE inhibitors for their in vivo efficacy [98] [Dranchak et al. (2023)]. The abovementioned advantages of *C. elegans* as a pre-clinical model organism, along with the established roles of nematode PDEs in many physiological and behavioral processes, position *C. elegans* to play an important role in elucidating disease mechanisms involving cyclic nucleotide signaling pathways as well as in identifying novel therapeutic treatments for human diseases.

## Figures and Tables

**Table 2:** Conservation of unanimous sites of human PDE catalytic domains with *C. elegans* PDEs. The 11 human PDE families (21 genes) were aligned with Jalview, trimmed to contain only the PDEase domain, then realigned with MAFFT (L-INS-i). Sequence numbering for the 17 unanimous sites is based on the human PDE4D4 sequence (Q08499-1). Then the six *C. elegans* PDE sequences were aligned with the human sequences to identify the residues corresponding to the human unanimous sites. The conservative substitution of alanine for serine in *C. elegans* PDE-2 is highlighted in red.

*C. elegans* PDE	PDE4D4 Residue # →	212	219	220	223	259	260	263	265	279	289	291	292	330	377	398	406	428
		**Y**	**H**	**N**	**H**	**H**	**D**	**H**	**G**	**A**	**E**	**H**	**H**	**T**	**D**	**E**	**E**	**Q**
1		Y	H	N	H	H	D	H	G	A	E	H	H	T	D	E	E	Q
2		Y	H	N	H	H	D	H	G	**S**	E	H	H	T	D	E	E	Q
3		Y	H	N	H	H	D	H	G	A	E	H	H	T	D	E	E	Q
4		Y	H	N	H	H	D	H	G	A	E	H	H	T	D	E	E	Q
5		Y	H	N	H	H	D	H	G	A	E	H	H	T	D	E	E	Q
6		Y	H	N	H	H	D	H	G	A	E	H	H	T	D	E	E	Q

**Table 3:** Summary of the functional diversity of *C. elegans* PDEs and their cyclic nucleotide signaling pathways. This table provides a synopsis of the experimental evidence presented in Section 3 for the physiological roles, cellular localization, and signaling pathway components for individual *C. elegans* PDEs. The information on cellular localization is restricted to the determination of the cells in which PDE activity is directly or indirectly measured in vivo or ex vivo; for the cellular localization of *C. elegans* PDE gene expression, readers are directed to Wormbase [16]. Signaling pathway components in which a *C. elegans* PDE has been experimentally determined to participate are abbreviated as follows: AC, adenylyl cyclase; GC, guanylyl cyclase; GPCR, G-protein coupled receptor; Gα, heterotrimeric G-protein α-subunit; Gβ, heterotrimeric G-protein β-subunit; PKA, cAMP-dependent protein kinase; PKG, cGMP-dependent protein kinase; CNGC, cyclic nucleotide-gated channel. When the *C. elegans* gene has been identified, the protein name is provided in parentheses.

*C. elegans* PDE	Physiological Role	Cellular Localization	Signaling Pathway Components	Section
PDE-1	chemosensation: CO_2_	BAG neurons	GC (GCY-9), Gα, CNGC (TAX-2, TAX-4)	Section 3.1.1
	chemosensation: O_2_	PQR neurons	GC (GCY-35), CNGC (TAX-2, TAX-4)	
	thermosensation	AFD and ASJ neurons	GC (GCY-8, -18, -23), CNGC (TAX-2, TAX-4)	
PDE-2	thermosensation	AFD neurons	GC (GCY-8, -18, -23), CNGC (TAX-2, TAX-4)	Section 3.2.1
	developmental progression	N.D.	GC (GCY-12); PKG (EGL-2)	
PDE-3	thermosensation	ASJ neurons	GC, Gα (GOA-1, GPA-1, GPA-3), CNGC (TAX-2, TAX-4)	Section 3.3.1
	phototransduction	ASJ neurons	GC (DAF-11, ODR-1), GPCR (LITE-1), Gα (GOA-1, GPA-3), CNGC (TAX-2, TAX-4)	
	chemosensation: multiple odorants	AWC neurons	GC (ODR-1, DAF-11), GPCR, Gα (ODR-3, GPA-2), PKG (EGL-4), CNGC (TAX-2, TAX-4)	
PDE-4	mechanosensation	ASH neurons	GPCR (DOP-1), AC (ACY-1), Gα (GSA-1), PKA (KIN-2)	Section 3.4.1
	locomotion	motor neurons	AC (ACY-1), Gα (GSA-1), PKA (KIN-2)	
	developmental progression	N.D.	AC (ACY-1), Gα (GSA-1), PKA (KIN-2)	
	neuronal development	PLM neurons	AC, Gα (GSA-1), PKA (KIN-2)	
PDE-5	thermosensation	AFD neurons	GC (GCY-8, -18, -23), CNGC (TAX-2, TAX-4)	Section 3.5
	chemosensation: multiple odorants	AWB neurons	GC (ODR-1, DAF-11), GPCR, Gα (GPA-3, ODR-3), CNGC (TAX-2, TAX-4, CNG-3)	
PDE-6	developmental progression	gonadal sheath cells; spermathecae	AC (ACY-4), Gα (GSA-1), Gβ (GPB-1), PKA (KIN-1, KIN-2)	Section 3.6

## Data Availability

The information used to generate the phylogenetic tree in Figure 2 is provided in the Supplementary Materials.

## Supplementary Materials

The following supporting information can be downloaded at: https://www.mdpi.com/article/10.3390/cells14151174/s1, Supplementary Materials (summary of the files used in creating Figure 2, including additional details not mentioned in the Figure 2 legend); Pdease_domain_brafl-filt_gt200_clust99.fasta (a list of the trimmed, filtered, and clustered pdease regions used in the final alignment in FASTA format); pde_tree_draft5-brafl-filt.aln (the output from Clustal Omega that is used as the input to IQ-TREE); pde_tree_draft5_renamed_brafil-filt.contree (the untransformed consensus tree output from IQ-TREE); pde_tree_draft5_key.csv (a list of the Uniprot entries used in the final analysis, and a second column corresponding to the truncated name used to represent the entry in the tree); pde_tree_draft5.nwk (the final version of the tree seen in Figure 2, complete with re-rooting, rotations, and name simplification).

## Abbreviations

The following abbreviations are used in this manuscript:

PDE, 3′,5′-cyclic nucleotide phosphodiesterase; AC, adenylyl cyclase; GC, guanylyl cyclases; GAF domain, protein domain first identified in cGMP-specific phosphodiesterases, Adenylyl cyclases and the bacterial FhlA protein; CaM, calmodulin; UCR, Upstream Conserved Region regulatory element found in the PDE4 enzyme family; REC, Receiver domain; PAS, Per-Arnt-Sim domain; IBMX, 3-Isobutyl-1-methylxanthine. Nomenclature: *C. elegans* PDE genes are denoted in lower-case italics (e.g., *pde-1*), whereas the *C. elegans* PDE gene product is shown as upper-case letters with a dash preceding the family number (PDE-1) to distinguish the nematode PDE from its mammalian ortholog (PDE1).
