# Supplementary material for: Roles of Cyclic Nucleotide Phosphodiesterases in Signal Transduction Pathways in the Nematode Caenorhabditis elegans
Source: Cells. 2025 Jul 30;14(15):1174. doi: 10.3390/cells14151174 (PMC12346295; doi:10.3390/cells14151174)
Supplement: Supplementary file 1 [file cells-14-01174-s001.zip › Supplementary Materials.pdf]

Supplementary Materials for

Roles of cyclic nucleotide phosphodiesterases in signal trans-duction pathways in the nematode *Caenorhabditis elegans*

Kranti K. Galande and Rick H. Cote

Below is the supporting information used in the process of creating the phylogenetic tree shown in Fig. 2 in the main text of the paper. The Supplementary Materials consist of the following files:

- **Pdease_domain_brafl-filt_gt200_clust99.fasta**
  - a list of the trimmed, filtered, and clustered pdease regions used in the final alignment in FASTA format.
- **pde_tree_draft5-brafl-filt.aln**
  - the output from Clustal Omega that is used as the input to IQ-TREE
- **pde_tree_draft5_renamed_brafil-filt.contree**
  - the untransformed consensus tree output from IQ-TREE.
- **pde_tree_draft5_key.csv**
  - a list of the Uniprot entries used in the final analysis, and a second column corresponding to the truncated name used to represent the entry in the tree.
- **pde_tree_draft5.nwk**
  - the final version of the tree seen in the publication, complete with re-rooting, rotations, and name simplification.

Additional details relevant to reproducing Fig. 2 that were not mentioned in the Fig. 2 caption are below:

- Some BRAFL entries (C3Z0J8_BRAFL, C3ZNK9_BRAFL, C3ZS98_BRAFL, A0A9J7HQW6_BRAFL*, A0A9J7HNR0_BRAFL*) were filtered from the final alignment and tree as they corresponded to alternative splicing of other entries included in the tree* or were identified as artifacts of sequencer error with suppressed entries in NCBI. These entries were filtered only after noting negligible branch lengths with another BRAFL entry in the original tree. The alignment and tree construction steps were re-run without these entries to create the final tree.
